# Management of Recurrent Undescended Testis: An Observational Study

**DOI:** 10.7759/cureus.100896

**Published:** 2026-01-06

**Authors:** Manish Rajput, Anand Panday, Gurmeet Singh, Rahul K Rai, Nitin Pant, Jiledar Rawat

**Affiliations:** 1 Pediatric Surgery, King George's Medical University, Lucknow, IND

**Keywords:** orchiopexy, recurrent udt, redo orchiopexy, surgery, undescended testis

## Abstract

Introduction: Recurrent undescended testis (UDT) may result from the failure of the orchiopexy. Despite the high success rate of primary orchiopexy, complications may still occur. This study presents our experience of managing recurrent UDT.

Materials and methods: This retrospective observational study was conducted from January 2015 to January 2025. The hospital records of all patients presenting with recurrent UDT were reviewed and analyzed. Data were extracted from patient files, operative registers, and electronic medical records. The parameters assessed included demographic characteristics, details of prior orchiopexy, intraoperative findings during redo surgery, and postoperative outcomes on follow-up.

Results: During the 10-year study period, 558 orchiopexies were performed. Of these, seven (1.25%) were redo orchiopexies. Five of these were operated on elsewhere -three by pediatric surgeons and two by general surgeons. The mean age of the patients was 7.6 years. All patients had a testis in the inguinal canal. In follow-up, testicular atrophy was noted in one patient. The testicular size appeared to be smaller compared to the opposite testis in five patients.

Conclusion: Recurrent UDT, although uncommon, poses a significant surgical challenge. Identifying the etiological factors, if present, is imperative. Successful management depends on careful dissection, preservation of vascular integrity, and tension-free subdartos placement of the testis. The overall outcome appears to be good in the hands of experienced professionals.

## Introduction

Undescended testis (UDT), also known as cryptorchidism, is one of the most common congenital anomalies in male children, affecting approximately 1-3% of full-term and up to 30% of preterm male neonates [1,2]. The condition results from failure of the testis to descend into the scrotum and may present unilaterally or bilaterally. Early surgical correction by orchiopexy is recommended to optimize spermatogenic potential, reduce the risk of malignancy, and facilitate examination [1-3].

The success rate of primary orchiopexy now exceeds 95% due to advancements in surgical techniques and a deeper understanding of testicular vascular anatomy [1,3,4]. However, complications such as atrophy or recurrent UDT may happen. Despite advances, recurrent UDT remains a challenging entity. This study presents our experience of managing recurrent UDT. The aims of the study include our experience of managing this uncommon complication with an emphasis on safe testicular mobilization and possible causative factors.

## Materials and methods

This is a retrospective observational study conducted in the Department of Pediatric Surgery at King George's Medical University from January 2015 to January 2025. We followed the Strengthening the Reporting of Observational Studies in Epidemiology (STROBE) guidelines endorsed by the EQUATOR network for conducting this study.

The hospital records of all patients presenting with recurrent UDT were reviewed and analyzed. Data were extracted from patient files, operative registers, and electronic medical records. The parameters assessed included demographic characteristics, details of prior orchiopexy, intraoperative findings during redo surgery, and postoperative outcomes on follow-up. All patients who presented with recurrent UDT during the study period were included. All other patients, including retractile testis, were excluded.

Surgical technique

All redo orchiopexies were performed under general anesthesia by experienced pediatric surgeons. We followed the classical inguinal approach as described by Bevan [5]. Jones' pre-peritoneal approach for high testes was used if the testis was close to the deep inguinal ring [6].

The previous inguinal incision was utilized; however, it was extended laterally toward the anterior superior iliac spine (ASIS) to create a pristine space and increase the possibility of identifying the spermatic cord as it enters the deep inguinal ring.

Dense scar tissue and adhesions were carefully dissected using magnification to identify the cord structures while minimizing trauma. The processus vaginalis, if present, was ligated at the level of the deep inguinal ring. After releasing the peritesticular adhesions, the testis was mobilized adequately to achieve a tension-free position, and fixation was performed in a well-developed subdartos pouch within the scrotum using fine absorbable sutures.

Postoperative management included routine analgesia, scrotal support, and follow-up assessments at regular intervals to monitor testicular position and size. They were followed for at least two years on an outpatient basis.

## Results

During the 10-year study period, 558 orchiopexies were performed. Of these, seven (1.25%) were redo orchiopexies. However, five of these were operated elsewhere for the initial surgery. Of these, three were operated on by pediatric surgeons and two by general surgeons. Our departmental recurrence was 0.35% (Table 1).

**Table 1:** Details of patients presenting with recurrent undescended testis B/L: bilateral; Rt: right; Lt: left.

Age	Location	Site	Place of first operation	Initial presentation	Length of testis	Length of testis (1-year follow-up)	Length of opposite testis (1-year follow-up)
12 years	Inguinal canal	Right	University hospital	B/L	1.5 cm	1.5 cm	3 cm
7 years	Near deep ring	Right	Elsewhere	Right	1.6 cm	1.6 cm	2.5 cm
16 years	Inguinal canal	B/L	Elsewhere	B/L	Rt - 3 cm; Lt - 2 cm	Rt - 3 cm; Lt - 2 cm	-
7 years	Near deep ring	Left	University hospital	B/L	1 cm	1.5 cm	2.5 cm
2 years	Inguinal canal	Left	Elsewhere	Left	1x1 cm	Atrophic	1.8 cm
3 years	Near superficial ring	Left	Elsewhere	B/L	1.5 cm	1.5 cm	2 cm
6 years	Inguinal canal	Left	Elsewhere	Left	1 cm	1.5 cm	2.5 cm

**Figure 1:**
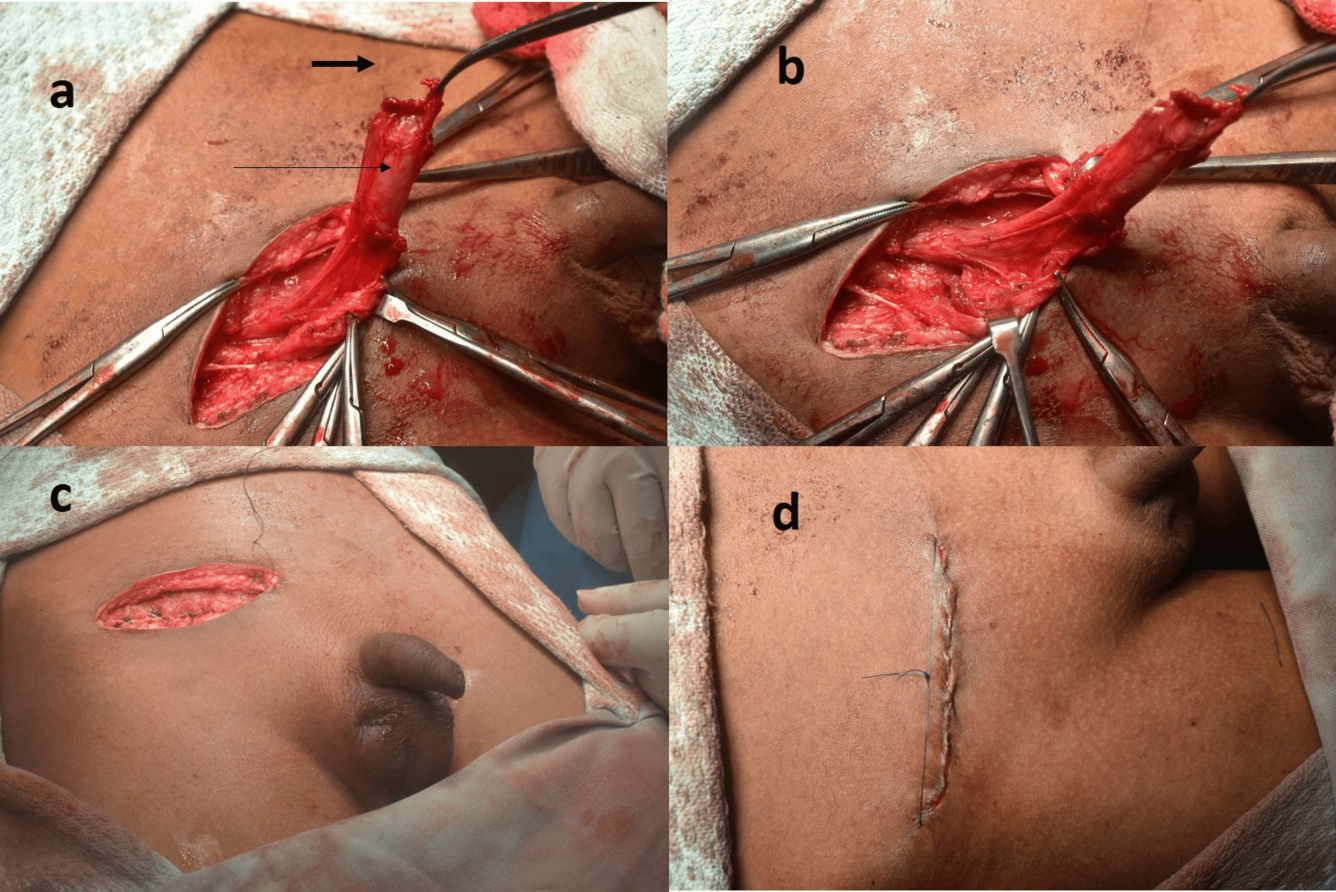
Operative steps in a recurrent undescended testis (a-d) Mobilization of the cord structures in a bilateral undescended testis patient operated previously at our center. The long arrow is pointing at the testis in (a) and the short, thick arrow is pointing at the previous scar line on the left side.

The incision was an inguinal crease incision in patients operated on by pediatric surgeons (Figure 1).

**Figure 2:**
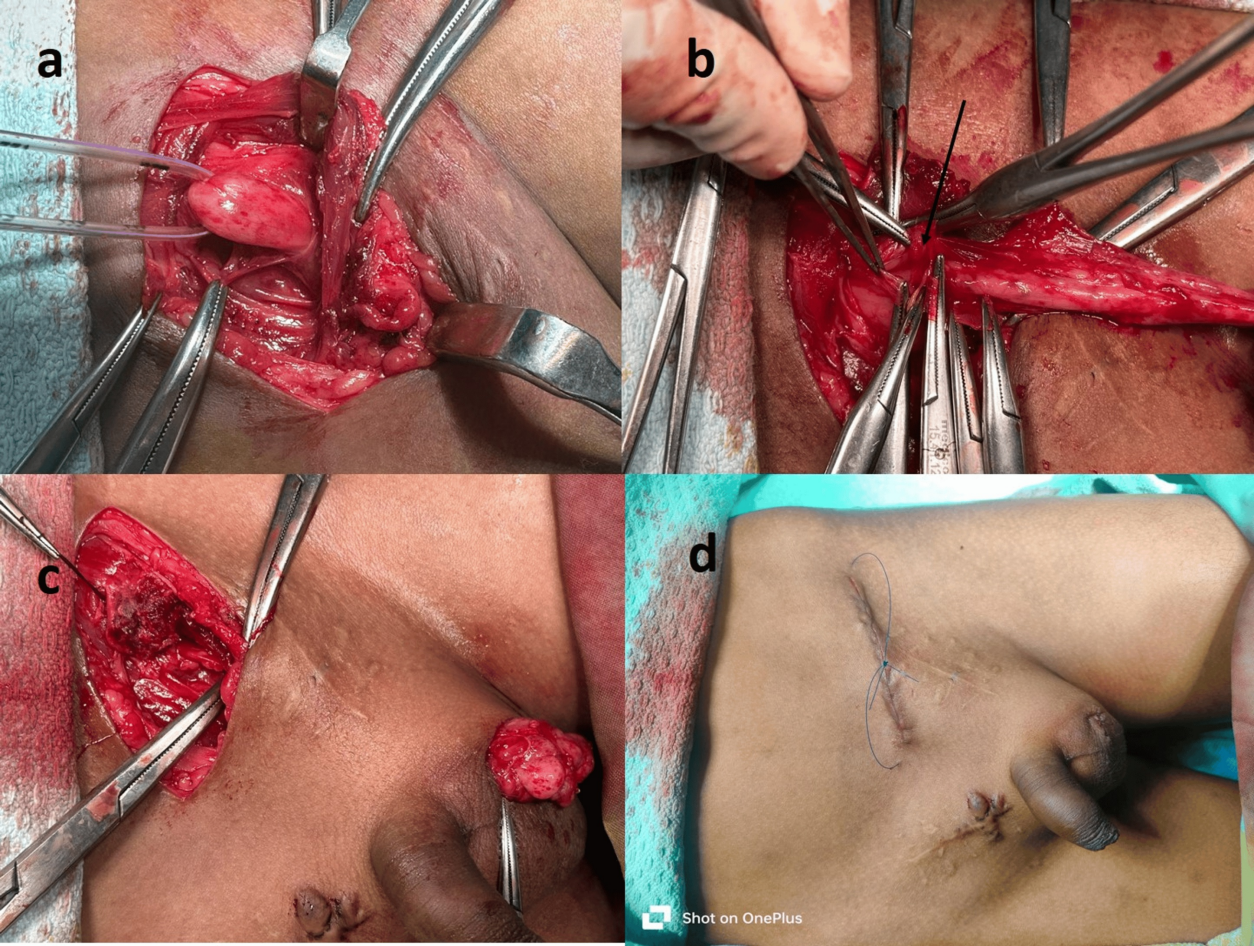
Redo orchiopexy in a patient operated on previously by a general surgeon (a-d) Redo orchiopexy in a previously operated bilateral undescended testis by a general surgeon. The cord structures are visible in (a). The arrow is pointing at the hernia sac in (b). The previous inguinal scars are visible in (d).

It was oblique, like adult hernia surgery in patients operated on by the general surgeons (Figure 2).

The mean age of the patients was 7.6 years (range 3-16 years). The median age was seven years. All patients had a testis in the inguinal canal. Four patients had bilateral UDT when they were operated on previously. One patient had a bilateral recurrence (Table 1). Four patients were operated on after two years of the first orchiopexy, while three patients were operated on after one year of the first surgery.

Dense peritesticular adhesions were present in all patients. It appeared that there was inadequate mobilization of the spermatic cord in three patients. In two patients operated on by the general surgeons, the hernia sac was not ligated. All patients had an uneventful postoperative recovery. Two patients underwent surgery using the Jones approach, while the remaining patients received a Bevan repair.

In follow-up, testicular atrophy was noted in one patient. This patient had a small testis at the time of redo orchiopexy. All other patients had testis present in the subdartos pouch. However, the size seemed to be smaller in comparison to the opposite testis in five patients. 

## Discussion

Recurrent UDT is an uncommon but significant complication of the orchiopexy procedure. Its management demands surgical precision and expertise. The recurrence rate is variable, with reports ranging from 0.2% to 10% [7]; however, recent data indicate a rate between 0.5% and 2% [2,3]. Our departmental recurrence was 0.3%, which falls within the reported rates.

Recurrence has been linked to insufficient cord mobilization, failure to repair the associated inguinal hernia, and lack of fixation of the mobilized gonad in a low intrascrotal position [7,8]. However, a recent study did not find a benefit from securing the testis in the subdartos pouch with an additional suture [9]. Thus, the role of testicular fixation needs further evaluation.

A key concern for redo orchiopexy is the presence of fibrosis and adhesions resulting from previous surgery. This may affect the safe mobilization of the cord structures. Bianchi operation, which describes testicular mobilization via scrotal approach, has been suggested for these patients. It has been suggested that it avoids the previous scarred site at the inguinal region. One study demonstrated a shorter operative time for redo orchiopexy using the Bianchi procedure compared to the inguinal approach [10]. However, it can be used only if the testis is located near the superficial inguinal ring, where it can be moved to the upper scrotal region [8].

If the inguinal approach is selected for a redo orchiopexy, various techniques are available to avoid injury to the cord structures. We used the Redman technique [11], as described in the Methods section, where we extended the previous incision laterally to enter the inguinal canal via a non-scarred space [11]. If the external oblique aponeurosis is adherent to the cord structures, a sleeve of it can be left attached to the cord structures [12]. To avoid traction on the cord structures, a strip of external oblique aponeurosis adherent to the cord structures can be fixed to the pubic bone or the gracilis tendon [13]. In our series, the external oblique aponeurosis was not severely adherent to the cord structures, and it could be separated with care.

It has been suggested that recurrent UDT is more common in bilateral UDT, as it reflects a more severe phenotypic expression [14]. However, it has not been statistically proven. It is of interest to note that both recurrences in patients operated on in our center had bilateral UDT. Although, we did not use laparoscopy in our series, as all recurrent UDTs were inguinal in position; however, it has been reported to be useful for recurrent non-palpable UDTs. A combined laparoscopy and inguinal approach has been suggested for such patients [15,16].

A unique situation was noted in our setup, as two patients were operated on by general surgeons. Their incisions were not proper, and the aesthetic appearance was less than satisfactory. This shows the importance of proper training in managing these specialized surgical conditions. 

The follow-up is an integral part of these patients. It has been shown that testicular volumes are lower compared to those of a normal testis [17]. Since most of our patients underwent their first surgery elsewhere, we are unsure of their initial testicular size. However, in our patients, the affected testis was smaller. Since four of them had a previous bilateral orchiopexy, we did not go for any comparative analysis of the testicular size of both sides. 

The limitation of this study is that it is a single-center study with a small sample size. Since recurrence is uncommon, a large sample size from a single center would be difficult. Additionally, we were unable to retrieve all details in a retrospective study.

## Conclusions

Recurrent UDT, although uncommon, poses a significant surgical challenge. Identifying the etiological factors, if present, is imperative. Successful management depends on careful dissection, preservation of vascular integrity, and tension-free subdartos placement of the testis. The overall outcome appears to be good in experienced hands.
